# Three-dimensional high-resolution anorectal manometry: A comparative pilot study with X-ray defaecography

**DOI:** 10.1097/MD.0000000000031682

**Published:** 2022-12-16

**Authors:** Qihong Liu, Wenyi Fang, Peilin Zhao, Yanqin He, HaiHua Gao, Xiao Ke

**Affiliations:** a The Second People’s Hospital Affiliated to Fujian University of Chinese Medicine, Fuzhou, China; b Fujian Province Traditional Chinese Medicine Spleen and Stomach Clinical Medicine Research Center, Fuzhou, China; c National Health Commission Traditional Chinese Medicine Spleen and Stomach Clinical Key Specialty, Fuzhou, China.

**Keywords:** paradoxical puborectalis syndrome, three-dimensional high-resolution anorectal manometry (3DHRAM), X-ray defecography

## Abstract

Three-dimensional high-resolution anorectal manometry (3DHRAM) is a new technique that can explore anorectal disorders and provide interesting topographic data for the diagnosis of pelvic floor disorders such as paradoxical puborectalis syndrome (PPS). Our object was to evaluate whether 3DHRAM can reliably diagnose PPS already diagnosed with X-ray defaecography, which is considered to be the gold standard.

All patients being tested in our department for dyschezia by 3D-HRAM and X-ray defecography were eligible for the study. The 3DHRAM results were compared with X-ray defecography. The sensitivity, specificity, and positive and negative predictive values were calculated for various 3DHRAM criteria to propose a diagnostic strategy for PPS.

Twenty-three patients presented with PPS on X-ray defaecography. On 3DHRAM, according to our diagnostic strategy, the kappa value was 0.706, with a positive predictive value of 71.88% [95% CI, 53.02–85.60], a specificity of 80.43% [95% CI, 65.62–90.13], a sensibility of 95.83% [95% CI, 76.98–99.78], and area under curve value was 0.922.

In this study, 3DHRAM was used to diagnose PPS with the same degree of reliability as X-ray defaecography, and we confirmed its use in the diagnosis of pelvic floor disorders. Further studies will be necessary to define classifications for these new anatomic data from 3DHRAM.

## 1. Introduction

Pelvic floor disorders are common, especially in women, with a prevalence ranging from 12% to over 20%.^[1]^ These disorders, often connected with Chronic constipation, may result in healthcare costs and a decreased quality of life. Detailed patient history and careful clinical examination remain the basis for diagnosis, but the choice of treatment usually requires further investigations. A variety of tests are now available, including defecography, rectal ultrasound, electromyography, anorectal manometry, and other tests. However, X-ray and MRI defecography remain the gold standard for assessing the morphology of pelvic floor disorders.^[2,3]^ Nevertheless, conventional defecography is radioactive, MRI defecography is expensive and not widely available, and both techniques take longer to examine and are often poorly tolerated and adhered to by patients.^[4]^ Three-dimensional high-resolution anorectal manometry (3DHRAM) is a new technique that can provide simultaneous analysis of pressures and the topography of the anal canal and provide physiological and morphological data simultaneously. 3DHRAM can not only diagnose anorectal dysfunction but also assess anal sphincter defects and pelvic floor disorders.^[5]^ Using this combination, a recent case report demonstrated that 3DHRAM can be used to diagnose paradoxical puborectalis syndrome (PPS).^[6]^

Our retrospective study aimed to determine whether 3DHRAM was able to reliably diagnose PPS, considering X-ray defecography as the gold standard.

## 2. Method

### 2.1 Patients

In this retrospective study, we analyzed the 3D-HRAM results received in our Gastrointestinal Dynamics Research Unit between January 2021 and September 2021. All patients must meet the diagnostic criteria for chronic constipation: less than 3 bowel movements per week; straining, difficult or incomplete bowel movements for at least 6 months. We included only patients who underwent 3DHRAM and X-ray defecography. In our center, these 2 checks are routinely performed on such patients. In our current practice, because of their specific expertise, 2 different operators performed the 2 procedures, but 3DHRAM was undertaken before X-ray defecography. Both investigations indicated constipation.

The inclusion criteria included: age above 18 years, dyschezia, and < 1 month between 3DHRAM and X-ray defecography. Exclusion criteria included: age under 18 years, history of anorectal surgery, inflammatory bowel disease, and organic pathology of the large bowel (including diverticulosis). Due to their specific expertise, 2 investigators performed both examinations. For all patients, a detailed clinical history was recorded, including age, gender, height, weight, duration of symptoms, etc. The severity of the predominant symptom, dyschezia was systematically evaluated using the Knowles Eccersley Scott symptom score.^[7]^

This study was approved by the Ethics Committee of the Second People’s Hospital of Fujian University of Traditional Chinese Medicine. All patients were fully informed of its purpose and their written consent was obtained.

### 2.2 3DHRAM

The test was carried out using the Mano Scan360TM 3D solid-state high-resolution anorectal manometry instrument from Medtronic, USA. All procedures were performed by a single experienced physician. All studies were carried out by 1 investigator with 5 years of experience with anorectal manometric examinations.

### 2.3 X-ray defecography

**Figure 1.**
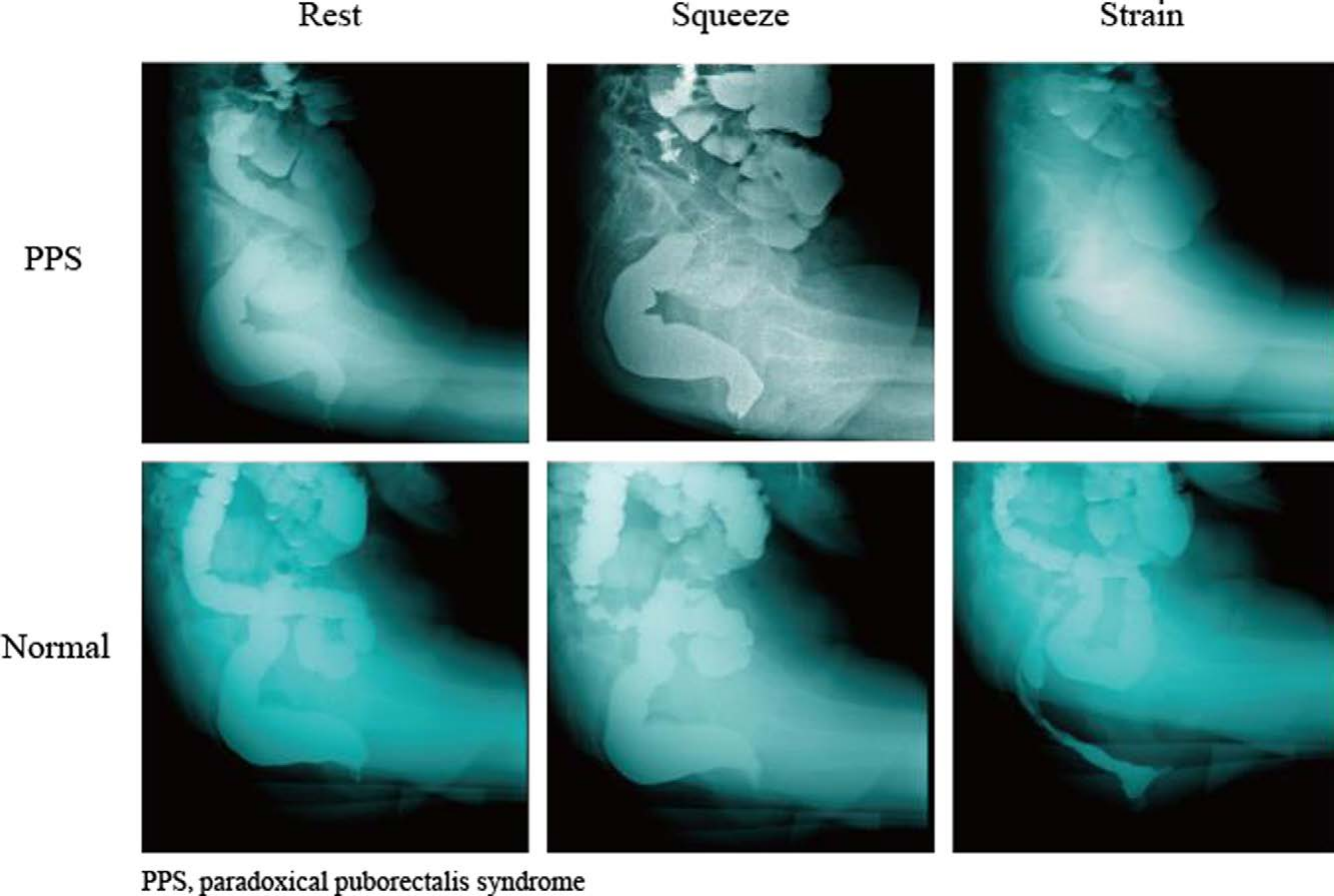
Comparison of X-ray defecography in normal healthy persons and PPS patients. (A) PPS: Puborectal muscle spasm in the simulated defecation when the anal rectal angle does not increase, still remain at about 90° or less, and more puborectal muscle spasm indentation. (B) Normal: The anorectal angle should be between 70° and 140° during simulated defecation.

X-ray defecography was performed using a simplified method described by Mahieu et al.^[8]^ Cleanse and wash the bowel before the examination to remove residual fecal matter from the intestine. After sufficient contrast filling of the rectum (400 mL of 75% sulfuric acid solution), the patients were asked to sit on a special commode, to adjust the height of the toilet seat so that it clearly shows the pubic symphysis, and then to perform simulated defecation, and images were taken under digital gastrointestinal machine fluoroscopy during resting, anal lifting, and forceful defecation, and the depth and length of the patient’s anorectal angle and puborectal muscle notch were recorded. This can be used to assess and measure the descent of the pelvic floor and to diagnose anterior rectal protrusion, rectal prolapse, or paradoxical puborectalis syndrome. A rectocele was defined as any anterior bulge of ≥ 2 cm outside the extrapolated line of the anterior rectal wall. Excessive perineal descent was defined as the descent of the anorectal angle to more than 3 cm below the pubococcygeal line on straining.^[9]^ Puborectal muscle spasm in the force row when the anal rectal angle does not increase, remains at about 90°or less, and more puborectal muscle spasm indentation (Fig. 1).

### 2.4 Statistical analysis

All demographic and patient-related data were recorded in SPSS 21.0 (SPSS Inc., Chicago, IL). Continuous variables are presented as means and standard deviations, and qualitative variables are presented as numbers and proportions. Comparisons of continuous variables between the 2 groups of patients were performed using the *t* test. The results of X-ray defecography were considered to be the gold standard technique in comparison with 3DHRAM. Comparisons of objective measurements and detection of pelvic floor anatomical and functional pathologies between 3DHRAM and X-ray defecography were reported as sensitivity, specificity, positive and negative predictive values, and Youden Index. The inter-test agreement was determined using the kappa statistic. The strength of agreement was graded as follows: 0 to 0.20, poor; 0.21 to 0.40, fair; 0.41 to 0.60, moderate; 0.61 to 0.80, substantial; and 0.81 to 1.00, almost complete to complete. Mapping receiver operating characteristics curve with the Graphpad Prism 8.0.1 software calculates the area under the curve line area under curve and its 95% CI.

## 3. Results

### 3.1 Patients

A total of 70 (65.71% female) constipation patients with a mean age of 52.69 ± 14.93 years were included in this study. The constipation symptoms were stressful defecation in 55 (78.57%), anal blockage sensation in 38 (54.29%), and sense of incomplete bowel movements in 43 (61.43%). All patient characteristics are presented in Table 1.

**Table 1:** Patient characteristics and X-ray defecography results.

Variables	Values
Gender	Men 24, Woman 46
Age (yr) (mean ± SD)	52.69 ± 14.93
Height (cm) (mean ± SD)	163.01 ± 7.13
Weight (kg) (mean ± SD)	58.43 ± 9.63
BMI (kg/m^2^) (mean ± SD)	21.94 ± 2.94
Duration of symptoms (yr) (mean ± SD)	8.71 ± 2.71
KESS (score) (mean ± SD)	22.01 ± 3.21
Symptoms	
Stressful defecation (n/%)	55/78.57%
Anal blockage sensation (n/%)	38/54.29%
Sense of incomplete bowel movements (n/%)	43/61.43%
X-ray defecography results	
Perineum descending	
·Number (n/%)	61/87.14
·Size (mm) (mean ± SD)	54.61 ± 13.75
Rectal protrusion	
Number (n/%)	31/44.29
Depth (mm) (mean ± SD)	21.48 ± 8.26
Paradoxical puborectalis (n/%)	24/38.57%
Sigmoid colon redundancy (n/%)	5/7.14%
Rectal mucosal prolapse (n/%)	3/4.29%

BMI = body mass index, KESS = Knowles Eccersley Scott symptom.

### 3.2 X-ray defecography

A PPS was present in 24 patients. All X-ray defecography findings are presented in Table 1.

### 3.3 3D High-resolution anorectal manometry

The results of the 3DHRAM are presented in Table 2. Using the X-ray defecography as the gold standard in Table 3, the kappa value was 0.706 (*P* < .001), the positive predictive value was 71.80% [95% CI, 53.02–85.60], the negative predictive value was 97.40% [95% CI,84.57–99.86], the sensitivity was 95.83% [95% CI, 76.98–99.78], the specificity was 80.43% [95% CI, 65.62–90.13].

**Table 2:** 3D high-resolution anorectal manometry results.

Variables	With PPS	Without PPS	*P*-value
Patients (n)	24	46	N/A
Mean resting pressure (mm Hg) (mean ± SD)	86.78 ± 27.89	72.73 ± 18.79	.015*
HPZ (cm) (mean ± SD)	3.95 ± 0.73	3.49 ± 0.88	.029*
Maximum squeeze pressure (mm Hg) (mean ± SD)	225.25 ± 77.33	178.44 ± 62.85	.008**
Duration of sustained squeeze(s) (mean ± SD)	16.02 ± 6.90	16.85 ± 5.49	.581
Residual anal pressure (mm Hg) (mean ± SD)	109.52 ± 39.23	69.70 ± 28.25	.000**
Relaxation rate (%) (mean ± SD)	−15.71 ± 24.86	9.91 ± 33.01	.001**
Intrarectal pressure (mm Hg) (mean ± SD)	39.00 ± 31.51	25.82 ± 21.25	.042*
Rectoanal pressure differential (mm Hg) (mean ± SD)	−70.49 ± 53.01	−43.98 ± 31.70	.011*
Mean volume of first sensation (mL) (mean ± SD)	48.33 ± 16.85	48.04 ± 16.14	.944
Volume for desire to defecate (mL) (mean ± SD)	89.17 ± 34.38	88.93 ± 26.69	.975
Maximum tolerable volume (mL) (mean ± SD)	133.33 ± 36.44	135.43 ± 37.10	.822
Recto-anal inhibitor reflex(mL)(mean ± SD)	23.75 ± 11.73	21.63 ± 12.65	.498
Dyssynergic pattern (n/%)			
Type I	4	4	.001**
Type II	17	15	
Type III	1	2	
Type IV	2	25	

HPZ = high pressure zone.

* *P* < .05.

** *P* < .01.

**Table 3:** Comparison of 3DHRAM to X-ray defecography.

3DHRAM	X-ray defecography		Kappa	Sensitivity	Specificity	PPV	NPV	AUC	Youden Index
	Positive	Negative							
Positive	23	9	0.706	0.958	0.804	0.718	0.974	0.922	0.678
Negative	1	37							

AUC = area under curve, NPV = negative predictive value, PPV = positive predictive value.

**Figure 2.**
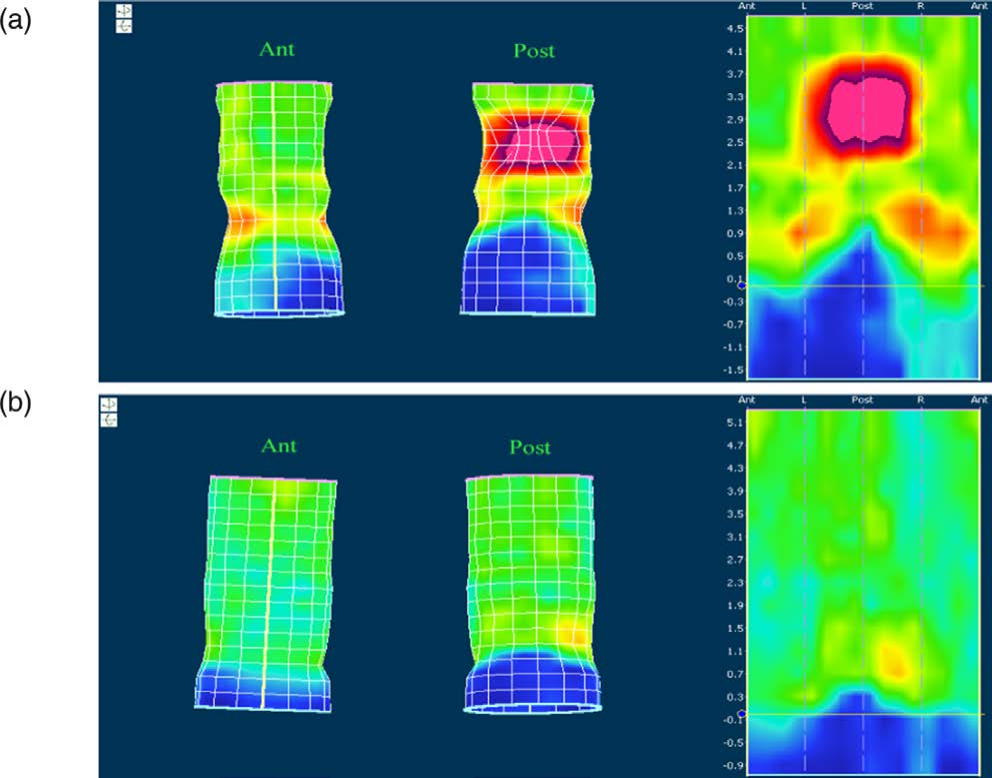
ROC curve.

Therefore, 3DHRAM and X-ray defecography are in sufficient agreement for the diagnosis of PPS and have a high diagnostic value. Because PPS is a benign lesion and its diagnosis is important before considering surgical options, specificity is the most relevant parameter. Therefore, we evaluated the pressure parameters of the puborectalis muscle during the 3DHRAM simulation of defecation, which was positioned approximately 2.5 to 4.0 cm from the anus, to establish a diagnostic strategy. We compared the pressure in the high-pressure area during simulated defecation in PPS patients with non-PPS patients and showed that the pressure was (174.37 ± 12.18 mm Hg) [95% CI, 149.11–199.63] significantly higher in PPS patients than in non-PPS patients (78.42 ± 5.40 mm Hg) [95% CI, 67.55–89.30] (*P* < .001). We plot the ROC curve by high-pressure values (Fig. 2), the area under curve value was 0.922 [95% CI, 0.861–0.983] with a Youden Index of 0.678. This suggests that the pressure value of the puborectalis muscle has a high diagnostic value for PPS.

## 4. Discussion

The puborectalis muscle starts at the pubic bone, moves backward around the anorectal junction, unites with the pubic bone, and forms a sling at the anorectal junction. At rest, a tense contraction of the puborectalis muscle pulls the anorectum forward, creating an anorectal angle of approximately 90°, which helps control bowel movements.^[11,12]^ During defecation, the puborectalis muscle relaxes to blunt the anorectal angle, thereby facilitating rectal evacuation. The term “paradoxical puborectalis syndrome” was first introduced by Wasserman in 1964.^[13,14]^ The most important finding of PPS is that the puborectalis muscle does not relax or have paradoxical contraction during the attempted evacuation, the pathophysiological mechanism of which remains incompletely understood. Outlet obstruction due to pelvic floor dysfunction accounts for more than 50% of cases of adult constipation.^[15]^ PPS is 1 of the common types of outlet obstruction constipation.

To our knowledge, the present study is the first to specifically evaluate the diagnostic capability of 3DHRAM for PPS, considering X-ray defecography as the gold standard. Although previous studies have shown that morphological data obtained using 3DHRAM can diagnose pelvic floor disorders, no one has specifically studied the diagnosis of PPS. Previous studies have shown that most of the resting pressure in the anal canal (70–80%) is associated with the internal anal sphincter and a small percentage with the external anal sphincter, and maximum squeeze pressure is associated with the external anal sphincter.^[16,17]^ In our study, the mean resting pressure and maximum squeeze pressure in the PPS patient group were significantly higher than the mean resting pressure in the non-PPS patient group in the resting state. This result suggests that resting pressure in patients with PPS is not only due to the internal and external anal sphincters but is also associated with incomplete relaxation or paradoxical contraction of the puborectalis muscle. It is widely believed that the length of the high-pressure zone reflects the length of the anal sphincter. In patients with PPS, prolongation of the anal canal and thickening of the puborectalis muscle can be found on physical examination. This was confirmed by our findings that the high-pressure zone length was also significantly longer in the PPS patient group than in the patients without PPS.

**Figure 3.**
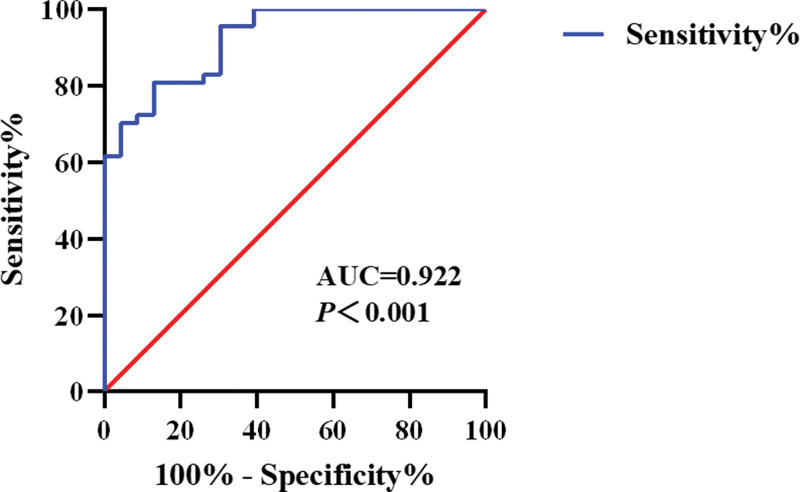
A: The 3HRAM in patients with PPS during simulated defecation, a characteristic purple high-pressure area in the distal posterior wall of anorectum in patients with PPS during simulated defecation. B: The 3HRAM in healthy adults during simulated defecation, indicates low pressure in the distal circumferential wall of anorectum in healthy adults during simulated defecation. Ant = anterior, PPS = paradoxical puborectalis syndrome, Post = posterior.

Our findings suggest that 3DHRAM can reliably diagnose PPS. All cases of PPS detected by X-ray defecography were also diagnosed by 3DHRAM. Conventional defecography remains the gold standard for the diagnosis of PPS.^[18]^ Of course, traditional anorectal manometry has not been more important than defecography in the past. Ger et al^[14]^ suggests that manometry may be the least painful, the most embarrassing, and the least influenced by psychological characteristics. However, manometry may also be the least valuable test, as rectal emptying cannot be assessed. Now, 3DHRAM has completely changed the previous conclusions. The pressure pattern in patients with PPS is completely different from that of healthy adults. In the 3D/2D pressure map (Fig. 3), a characteristic purple high-pressure area can be seen in the posterior rectal wall, which is not present in healthy adults. The purple high-pressure area showed the incomplete or paradoxical contraction of the puborectalis muscle. Purple high-pressure areas generally suggest an incomplete or paradoxical contraction of the puborectalis muscle, but we cannot determine this based on color alone. Because sometimes the pressure value is not high due to the setting of the pressure measurement parameters, it can cause inconsistency with the results of the fecal imaging. In this regard, we conducted a comparison of the pressure worthiness of the purple high-pressure region and initially arrived at a pressure reference value of 174.37 ± 12.18 mm Hg for the PPS, but further expansion of the sample size for comparison is also needed to arrive at an accurate reference parameter. Interestingly, 3D manometry in patients with PPS showed distinct areas of high pressure. These new pressure patterns detected by 3DHRM, in combination with pressure parameters, greatly increase the value of manometry in the diagnosis of PPS compared to conventional manometry techniques.

This study has some limitations. The first is the supine position of the patient in 3DHRAM, not the physiological defecation position, which may explain the difference between the measurement means. The diagnostic parameters of 3DHRM for PPS have not been determined yet. The second limitation is the small sample size. The third limitation is the lack of a normal control group. This study aimed to evaluate the accuracy of 3DHRAM for the diagnosis of PPS using X-ray defecography as the gold standard. Therefore, our next goal is to provide preliminary findings using this new technique and compare it with MRI defecography and rectal ultrasound.

In conclusion, this study demonstrates that 3DHRAM can reliably assess PPS. therefore, it is a suitable tool for the morphological assessment of pelvic floor disorders. It can also detect, localize and assess the extent and pressure of PPS, further demonstrating the value of manometry in diagnosis.

## Author contributions

Conception and design of this study: Qihong LIU, Wenyi FANG. Data generation, collection, assembly, analysis, and interpretation: Peilin Zhao, HaiHua GAO, Yanqin HE. Drafting or revision of the manuscript: Qihong LIU. Quality control and proofreading: Xiao KE. All authors have finally approved the publication of this edition.

**Conceptualization:** Xiao Ke.

**Data curation:** Peilin Zhao, HaiHua Gao.

**Formal analysis:** Yanqin HE.

**Writing – original draft:** Qihong Liu.
